# Cell-free DNA in plasma as an essential immune system regulator

**DOI:** 10.1038/s41598-020-74288-2

**Published:** 2020-10-15

**Authors:** M. Korabecna, A. Zinkova, I. Brynychova, B. Chylikova, P. Prikryl, L. Sedova, P. Neuzil, O. Seda

**Affiliations:** 1 Institute of Biology and Medical Genetics, First Faculty of Medicine, Charles University and General University Hospital in Prague, Albertov 4, 128 00 Prague, Czech Republic; 2 Department of Anthropology and Human Genetics, Faculty of Science, Charles University, Prague, Czech Republic; 3 First Faculty of Medicine, Institute of Pathological Physiology, Charles University, Prague, Czech Republic; 4 Department of Microsystem Engineering, School of Mechanical Engineering, Northwestern Polytechnical University, Xi’an, People’s Republic of China; 5 CEITEC, Brno University of Technology, Brno, Czech Republic

**Keywords:** Cell biology, Genetics, Immunology

## Abstract

The cell-free DNA (cfDNA) is always present in plasma, and it is biomarker of growing interest in prenatal diagnostics as well as in oncology and transplantology for therapy efficiency monitoring. But does this cfDNA have a physiological role? Here we show that cfDNA presence and clearance in plasma of healthy individuals plays an indispensable role in immune system regulation. We exposed THP1 cells to healthy individuals’ plasma with (NP) and without (TP) cfDNA. In cells treated with NP, we found elevated expression of genes whose products maintain immune system homeostasis. Exposure of cells to TP triggered an innate immune response (IIR), documented particularly by elevated expression of pro-inflammatory interleukin 8. The results of mass spectrometry showed a higher abundance of proteins associated with IIR activation due to the regulation of complement cascade in cells cultivated with TP. These expression profiles provide evidence that the presence of cfDNA and its clearance in plasma of healthy individuals regulate fundamental mechanisms of the inflammation process and tissue homeostasis. The detailed understanding how neutrophil extracellular traps and their naturally occurring degradation products affect the performance of immune system is of crucial interest for future medical applications.

## Introduction

The utilization of the DNA localized outside the cells by bacteria was reported by Avery et al. in 1944^1^. The existence of cell-free DNA (cfDNA) in human blood was first reported in 1948^2^. Initially, the biological significance of cfDNA was studied especially by Russian scientists using blood transfusion in animals, the first grafting experiments in plants were performed and documented by Stroun et al. in 1963^3^. All these experiments documented the horizontal transmission of genetic material and were evaluated mainly from evolution point of view^4^. The discovery of increased concentration of cfDNA in plasma of patients with the autoimmune disorder systemic lupus erythematosus (SLE)^5^ as well as in plasma of patients diagnosed with cancer represented the initial clinical studies. The discovery of cfDNA of fetal origin circulating in the plasma of pregnant women^6^ triggered an avalanche of interest in cfDNA research and subsequently led to the establishment of non-invasive prenatal diagnostics. The investigation of cfDNA from plasma of patients with cancer revealed the presence of tumor mutations, resulting in the concept of *liquid biopsy*^7^. Nowadays, clinicians study intensively the plasma cfDNA for prenatal testing^8^, as well as to monitor therapy efficiency in oncology^9^ and transplantology^10^.

It is accepted that the cfDNA in plasma originates from hematopoietic cells and contains sequences of entire genomes. The cfDNA is presented in the form of short fragments corresponding to DNA length wrapped around one nucleosome or its multiples^11^. The cfDNA molecules circulate in complex together with at least histones and other scantly characterized compounds^12^, but the detail structures of these complexes are not well studied. The standard conventional DNA isolation procedure may destroy or modify these vital cfDNA complexes, thus change their biological behavior.

Previously, cfDNA clearance in plasma was studied by monitoring the therapeutic effects of venous DNase I administration in mouse models of SLE as well as in patients with SLE^13,14^. These studies reported that the cfDNA clearance rate is linked to the DNase I concentration. Also it was found that the DNase I had elevated concentrations in the blood plasma of patients developing sepsis after major trauma^15^. Neutrophil extracellular traps (NETs) containing entire nuclear DNA of neutrophils in association with bactericidal components are released to catch and destroy the infectious microorganisms. These NETs are subsequently cleared from the circulation by DNase I and their insufficient clearance can result in occlusion of blood capillaries, leading to impaired microcirculation, enzymatically damaging tissues and further progression of inflammation^15^. The elevated formation of NETs was reported in most comorbidities worsening the clinical course of COVID-19^16^. The hyperactivated neutrophils and monocytes-macrophages are the usual initiators of the cytokine storm responsible for the most serious consequences of coronavirus SARS CoV-2 infection^17^. Bacteria and protozoa are able to convert the NETs using their own enzymes into the DNA degradation product such as deoxyadenosine which is toxic for macrophages and causes their apoptosis^18,19^.

Despite all these findings and the massive cfDNA-based diagnostic technique developments, there are surprisingly few studies focused on the fundamental biological function of cfDNA in healthy individuals. One study treated human monocytes either with the plasma of dialyzed patients or healthy individuals, both containing cfDNA. Only the plasma from dialyzed patients stimulated the production of pro-inflammatory interleukin IL 6 in target cells^20^. It was also revealed that the cfDNA isolated from individual tumor cell lines or complex tumors injected into animal blood circulation caused tumor transformation, referred to as genometastasis^21^. The biological character and function of unmethylated fetal cfDNA was explored and the increased proportion of fetal cfDNA in maternal circulation during pregnancy was found^22,23^.This fetal cfDNA stimulated a maternal immune response against the placenta, resulting in the proper timing of labor^22^.

The influence of cfDNA on human macrophages was tested using isolated cfDNA from various blood products with different storage time to emulate the conditions immediately after transfusion. The cells were cultivated in the presence of calf serum bearing its own cfDNA. The study found increased expression of genes involved in the innate immune response (IIR), including chemokines and their receptors in macrophages, and concluded that cfDNA contained in the stored blood products might interfere with the immune system of transfusion recipients^24^. The authors reported the elevated expressions of CXCL8 and DDIT3 in experiments, but the results may be affected by the presence of calf serum in all experiments.

This raises the following question: *What is the fundamental role of cfDNA in healthy organisms*? We assumed that we could find it by exposing identical cell lines to plasma with and without cfDNA and excluding any other factors, such as calf serum or potential damage of cfDNA complexes by isolation procedure.

## Results

We performed all stimulatory experiments using the THP1 cell line as a representative of primary human monocytes^25^ to show the fundamental role of cfDNA in healthy organisms. The experiments were conducted in duplicates using plasma containing cfDNA (NP) and the reference one with cfDNA removed by DNase (TP) to recognize the effect of plasma cfDNA on transcriptome and proteome of monocytes. We used native human plasma samples obtained from healthy volunteers with no animal serum addition to the cultivation medium in order to avoid the presence of uncharacterized animal cfDNA and DNases in the experiments.

**Figure 1:**
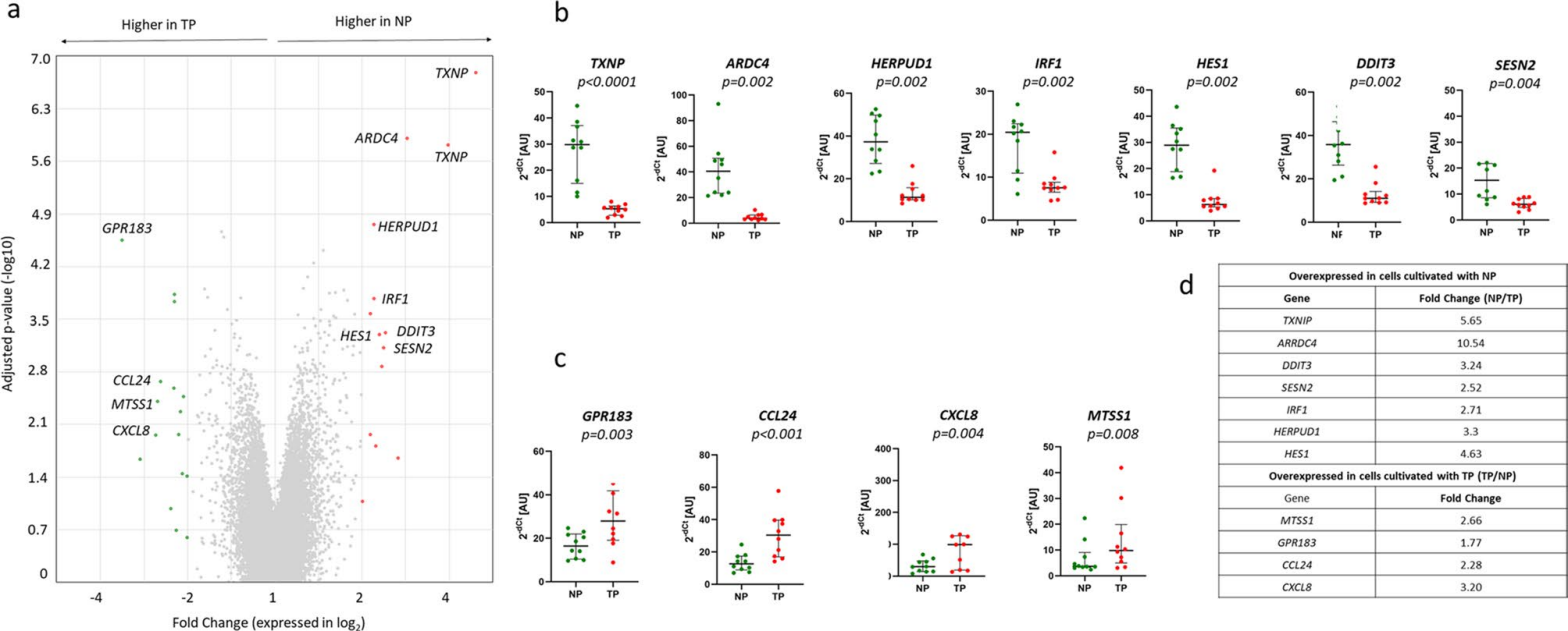
Differentially expressed genes in cells treated with native plasma (NP) and with DNAase treated plasma (TP). (**a**) Comparison of gene expression in THP1 cells cultivated with NP versus TP (GeneChip Human Gene 2.1 ST Array Strip technology, Supplementary Data Table 1). Analysis performed and graph derived using Transcriptome Analysis Console 4.0 (https://www.thermofisher.com/cz/en/home/life-science/microarray-analysis/microarray-analysis-instruments-software-services/microarray-analysis-software/affymetrix-transcriptome-analysis-console-software.html). (**b**) Genes overexpressed in THP1 cells treated with NP—results of validation qPCR experiments normalized to the PGK1 gene. AU = arbitrary unit, graphs generated using GraphPad Prism 5.00.288 software, https://www.graphpad.com. (**c**) Genes overexpressed in THP1 cells treated with TP*—*results of validation. (**d**) Results of validation qPCR experiments given as fold changes.

In the discovery phase, we used six plasma samples, searching for differences in transcriptomes related to treatment with NP and TP using GeneChip Human Gene 2.1 ST Array Strip (Thermo Fisher Scientific). We detected significant differences (Fig. 1a; Supplementary Table 1), which were further validated using single-target quantitative PCR (qPCR) and another ten plasma samples (Fig. 1b–d).

**Table 1:** Results of analysis performed using the Reactome database. (a) Over-represented pathways for genes upregulated in THP1 cells treated with NP and (b) cells treated with TP; (c) Over-represented pathways for proteins upregulated in THP1 cells treated with TP (FDR false discovery rate with Benjamini–Hochberg adjustment).

Process	Pathway name	FDR	Genes
**(a) Pathways over-represented in cells treated with native plasma containing cfDNA (NP)**			
Unfolded protein response (UPR) maintains immune system homeostasis	ATF4 activates genes in response to endoplasmic reticulum stress	1.28 × 10^−6^	*DDIT3, HERPUD1*
	PERK regulates gene expression	1.52 × 10^−6^	*DDIT3, HERPUD1*
	Unfolded Protein Response	1.38 × 10^−6^	*DDIT3, HERPUD1*
	ATF6 (ATF6-alpha) activates chaperone genes	8.90 10^−4^	*DDIT3*
Constitutive tonic NOTCH signaling	RUNX3 regulates NOTCH signaling	8.16 × 10^−6^	*HES1*
	NOTCH1 Intracellular Domain Regulates Transcription	3.00 × 10^−3^	*HES1*
	Signaling by NOTCH1	5.00 × 10^−3^	*HES1*
Regulation of RNA polymerase II transcription	Generic transcription pathway	9.49 × 10^−4^	*DDIT3, HES1, SESN2, TXNIP*
	RNA polymerase II Transcription	1.00 × 10^−3^	*DDIT3, HES1, SESN2, TXNIP*
**(b) Pathways over-represented in cells treated with native plasma without cfDNA (TP) as the consequence of** ***CXCL8* overexpression**			
Innate immune response activated by UPR	ATF4 activates genes in response to endoplasmic reticulum stress	8.32 × 10^−4^	
	PERK regulates gene expression	8.32 × 10^−4^	
Interleukin signaling	Interleukin-10 signaling	2.00 × 10^−3^	
	Interleukin-1 and interleukin-13 signaling	6.00 × 10^−3^	
Cellular senescence	Senescence associated secretory phenotype	2.00 × 10^−3^	
**(c) Pathways over-represented in cells treated with native plasma without cfDNA (TP)**			
Complement activation	Complement cascade	1.34 × 10^−10^	SERPING1, CFHR5, C7, C9, CFD, C1QB, C1QA, C1QC, C1S, CFH, C1R, C4B, C3, C5, C6
	Regulation of complement cascade	1.34 × 10^−10^	SERPING1, CFHR5, C7, C9, C1QB, C1QA, C1QC, C1S, CFH, C1R, C4B, C3, C5, C6
	Terminal pathways of complement	5.39 × 10^−4^	C7, C9, C5, C6
	Initial triggering of complement	9.00 × 10^−3^	C1S, C1R, CFD, C1QB,C4B, C3, C1QA, C1QC

To differentiate between the effects of buffers alone and the effect of DNase Turbo treatment, we performed a set of experiments (Supplementary Fig. 1). The addition of activation buffer containing divalent cations is necessary for the activity of DNase Turbo in plasma originally treated with ethylenediaminetetraacetic acid (EDTA). The addition of this buffer alone led after incubation to the decrease of cfDNA in the plasma sample to 57.42% of its original level due to the activation of plasma endogenous DNase I. The incubation of plasma sample with this buffer and DNase Turbo resulted in the detection 7.07% of cfDNA original amount when measured after its isolation from plasma using qPCR.

In this set of stimulation experiments, we tested the effects of the complete procedure allowing the DNase Turbo activity in plasma and its subsequent removal but without the addition of DNase Turbo itself and compared the results with NP and TP samples. All these experiments were performed using a plasma sample obtained from an identical donor. We examined the expression of all validated genes and concluded that the activation of endogenous DNAse I was mostly sufficient to produce the basic differences which could be in some cases further strengthened with subsequent DNase Turbo treatment (Supplementary Fig. 1). The procedure serving for the removal of divalent cations was applied during the processing of all treated plasma samples to avoid the elevated concentrations of these ions in samples due to the DNase Turbo activation buffer addition. We received the results identical with the results of validation experiments in eight out of eleven examined genes as we explored the stimulatory capacity of only one differentially treated plasma sample. The expressions of *DIDT3* and *SESN2* were significantly elevated in cells treated with NP samples in validation experiments but decreased in cells treated with NP sample of this donor*.* The *CCL24* expression was significantly increased in cells stimulated with TP samples in validation study but elevated when treated with NP sample of this individual. The inconsistencies found in the expressions of *DDIT3, SESN2* and *CCL24* may thus reflect the individual variability deserving of further study.

We used the validation phase results to perform a direct comparison of signaling pathways activated in cells as a consequence of their treatment with NP or TP samples (Table 1a, b) using the database Reactome. This analysis demonstrated the critical importance of the presence/absence of the intact cfDNA for the expression profile of cells and regulatory pathways activation. This fact was also documented by the Reactome analysis of results obtained by mass spectrometry used for proteome examination of THP1 cells treated with NP or TP (Table 1c).

**Figure 2:**
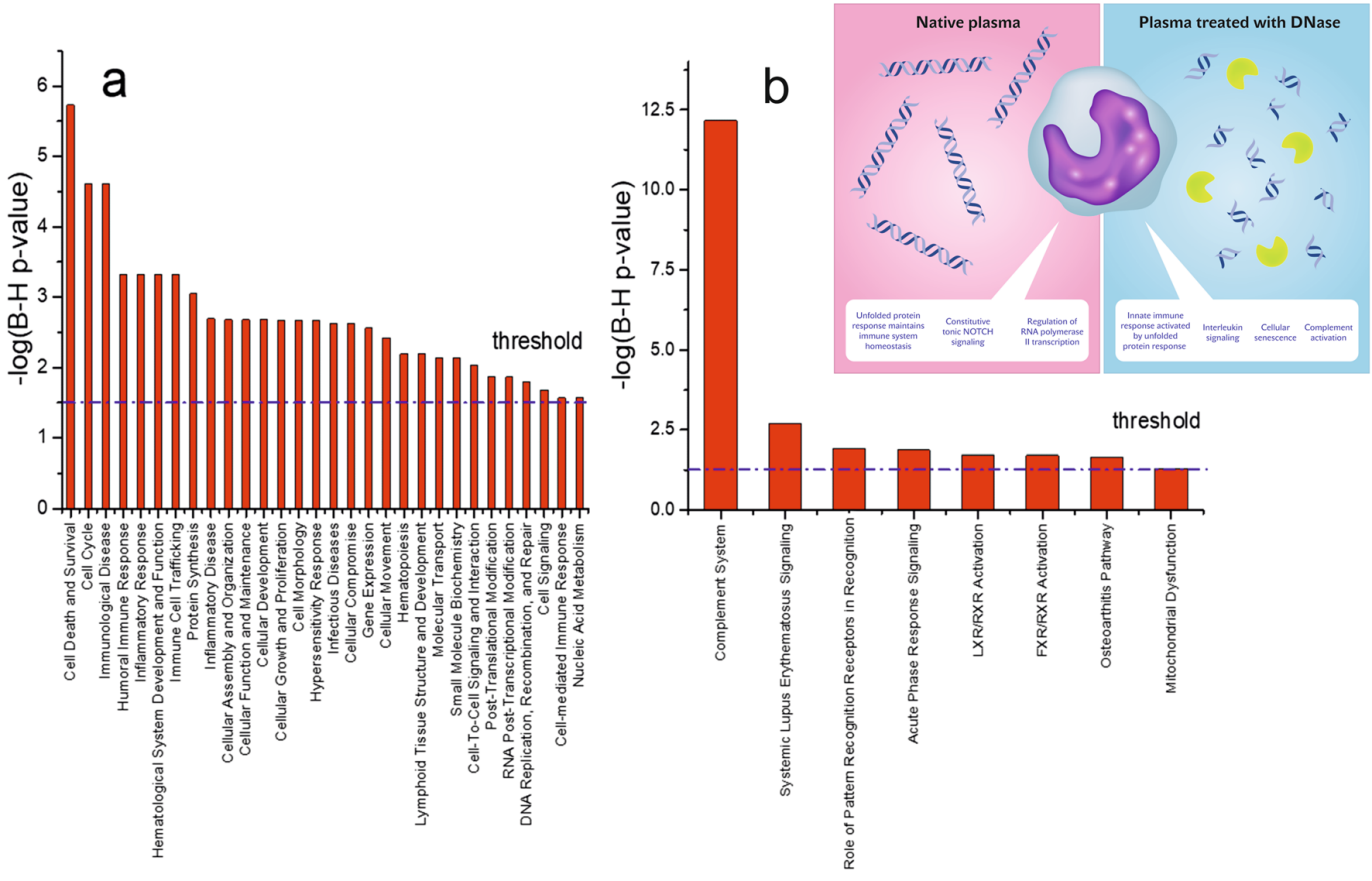
(**a**) Differentially expressed canonical pathways detected by IPA in THP1 cells treated with TP versus NP with a threshold value set to − 1.53, corresponding to the statistical significance level (α) value of 0.03. (**b**) Differentially expressed disease-specific pathways detected by IPA in THP1 with TP versus NP with a threshold value set to − 1.3, corresponding to the α value of 0.05. The analyses were generated through the use of IPA (QIAGEN Inc., https://www.qiagenbioinformatics.com/products/ingenuity-pathway-analysis) Inset: Differentially upregulated pathways in THP1 cells cultivated with native plasma and plasma treated with DNAse as detected using the Reactome database. LXR stands for liver X receptor, RXR for retinoid X receptor, and FXR for farnesoid X receptor.

**Figure 3:**
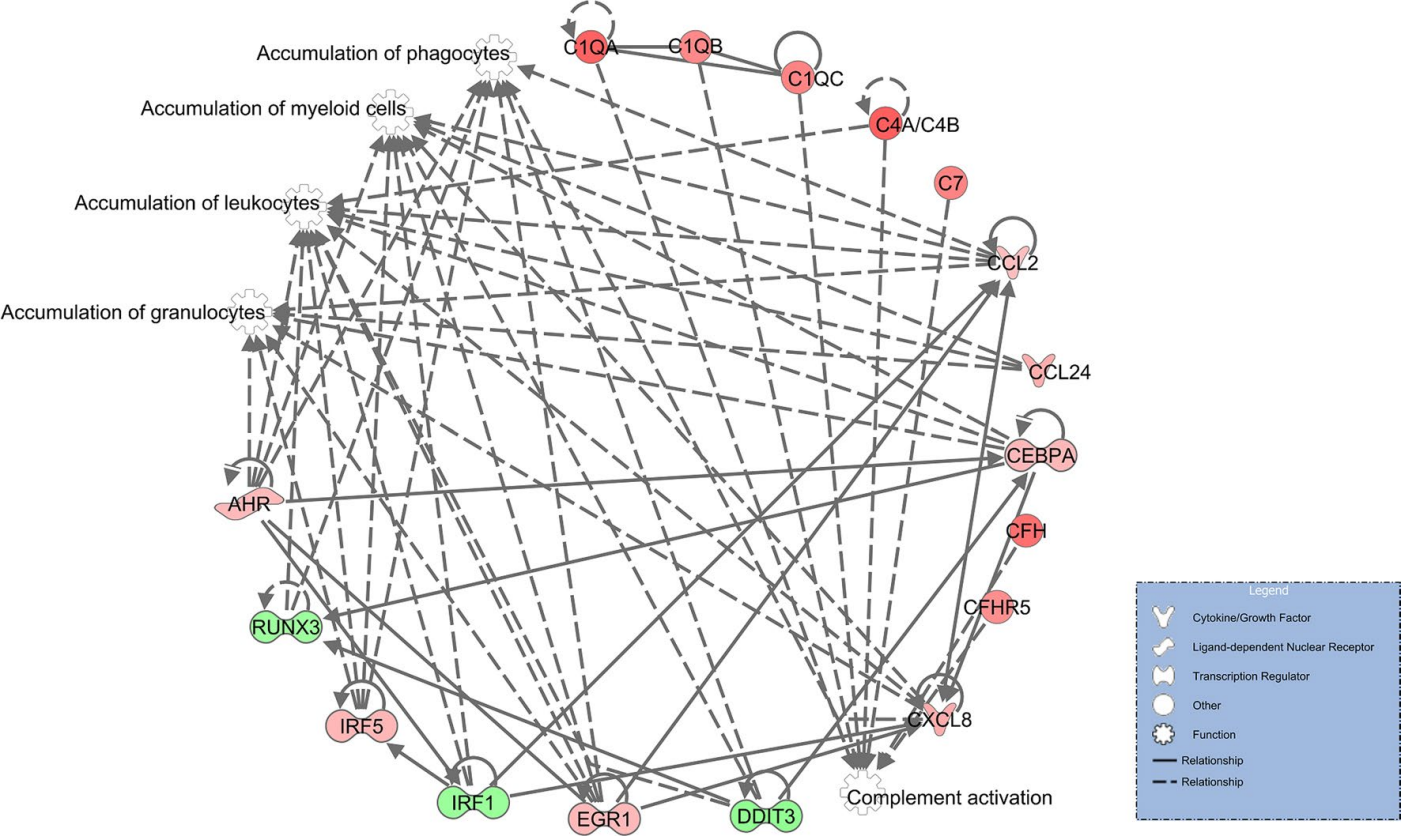
Target downstream biological pathways predicted by IPA (QIAGEN Inc., https://www.qiagenbioinformatics.com/products/ingenuity-pathway-analysis). The intensity of the red color mirrors the level of upregulation; the green color is used for downregulation.

The presented work documented that the cfDNA and its clearance in plasma is under physiological conditions indispensable for immune system performance. We demonstrated that monocytes in intact cfDNA presence (NP) upregulated the central pathways responsible for immune system homeostasis, especially notch signaling^26^ and unfolded protein response (Table 1a)^27^ while the degraded cfDNA in plasma (TP) resulted in upregulated *CXCL8* expression (Table 1b). This mRNA should translate into interleukin 8 (IL8), a pivotal protein involved in the direct activation of IIR^28^ and also regarded as a marker of cellular senescence^29^, but its elevated level was not found in proteomic analysis after our three-hour-long cultivation experiments. Nevertheless, we detected the upregulation of proteins contributing to the activation of complement (Table 1; Supplementary Table 2), confirming the inflammatory state of these cells. The software Ingenuity Pathways Analysis (IPA) was used to explore all obtained data (Supplementary Table 3). The IPA results (Fig. 2a, b) confirmed the findings of Reactome analysis (summarized in Fig. 2b inset). In THP1 cells cultivated with TP, multiple pathways involved in immune response were detected among significantly changed canonical pathways (Fig. 2a) and overrepresented disease-specific pathways (Fig. 2b). IPA also predicted the accumulation of granulocytes, leucocytes, myeloid cells, phagocytes, and complement activation as the consequence of events signalizing the presence of cells that are confronted mainly with degraded cfDNA (Fig. 3).

## Discussion

We developed and tested an experimental workflow which allowed us to compare the effects of cfDNA pool in native plasma and cfDNA in plasma degraded by endogenous DNase I and additionally with DNAse Turbo on the THP1 cells. The native plasma samples were fixed by EDTA as a potent indirect DNAse I inhibitor. In the EDTA treated plasma samples, the activity of the endogenous DNase I is completely stopped^30^. The activation of endogenous DNase I and the subsequent activity of DNase Turbo were allowed by addition of an activation buffer containing divalent cations to the EDTA treated plasma samples. To inactivate these divalent cations, the DNase Turbo inactivation procedure was applied according to the protocol provided by the manufacturer for the aqueous solutions of isolated RNAs. Our results are of course limited by the fact that we are working with such a complex sample as human blood plasma. It is not possible to design the experiments to exclude completely the influence of changing divalent cations concentrations^31,32^ during the entire experimental procedure, the presence of cfDNA hidden in plasma exosomes^33,34^ or in supramolecular complexes and on cell surfaces^35^. Nevertheless, when we quantified the cfDNA isolated from NP, from the sample exposed to the entire cfDNA removal procedure but without addition of DNase Turbo and from the TP sample, we found striking gradual decrease in cfDNA levels toward the last sample. The changes in expression profiles of selected validated genes were detectable after the decrease of cfDNA levels to 69.10% of its original native concentration as the result of endogenous DNAse I activity (Supplementary Fig. 1). Therefore we could speculate also about the role of cfDNA degradation products in the induction of these expression changes. The significance of differences in expression profiles of cells treated with NP and TP was statistically proven in validation experiments. We detected namely the differences in immune system regulatory pathways discussed in the next paragraphs.

The inflammatory response in mammalian cells is regulated by notch signaling pathways acting through four different notch receptors (NOTCH 1–4) transducing extracellular signals^26^; the NOTCH 1 is involved in myeloid lineage differentiation leading to monocytes ^36^. The constitutive tonic activity of notch signaling pathways was described in non-activated immune cells^26^. We found that the monocytes treated with NP expressed *HES1* mRNA by  ≈ 4.63 × more than the ones treated with TP (Fig. 1d). This elevated expression documents the notch pathways’ activity in these cells. The pathways leading from toll-like receptors (TLRs) may provide additional signals to the notch ligands^26^. In primary macrophages, it has been shown that the notch target genes, like *HES1,* can also be induced exclusively by TLR stimulation^37^. In such a model, the presence of cfDNA itself could ensure the constitutive tonic notch signaling via TLR9 as a receptor specialized in DNA sensing.

Different types of cellular stress lead to unfolded protein accumulation in the lumen of the endoplasmic reticulum. This accumulation activates signal-transduction cascade known as unfolded protein response (Table 1a). It has been demonstrated that this cascade plays the central role in the modulation of immune system functions regarding both innate and adaptive responses^27^.

In our experiments, the expression of *SESN2* (sestrin 2) is upregulated in cells treated with NP (Fig. 1b). *SESN2* is well-known as a stress-inducible protein suppressing inflammasome activation by the induction of mitophagy. *SESN2* plays a crucial role in this unique regulatory mechanism of mitophagy activation which is pivotal for the maintenance of immunological homeostasis and protection from sepsis^38^.

We found the increased expression of *ARRDC4* and *IRF1* genes in cells treated with NP (Fig. 1b) additionally to the genes involved in the pathways detected by Reactome (Table 1). The role of arrestin domain-containing 4 (ARRDC4) in IIR was recognized but only partially understood^39^. This gene is transcribed in monocytes in healthy individuals; after a viral infection, its levels were increased and correlated with concentrations of interleukins in serum. Interferon regulatory factor-1 (*IRF1*) is a transcription factor expressed at low levels in immune system cells and induced by different cytokines. It controls the transcription of its target genes in different types of immune cells^40^.

Cultivation of THP1 cells with TP led to the change of expression pattern. Apart from increased *CXCL8* expression, we found upregulated expression in *MTTS1*, *GPR1,* and *CCL24* (Fig. 1c). Metastasis suppressor-1 (MTSS1) was originally identified as a metastasis suppressor in the carcinoma cell line. Nowadays, it is known that this multifunctional cytoskeleton scaffold protein regulates the cytoskeleton dynamics and inhibits cell migration^41^. G protein-coupled receptor 183 (GPR183) is a member of the signaling pathway leading to repression of notch signaling^42^. *CCL24* codes for eotaxin 2, which is produced by activated monocytes and attracts lymphocytes, basophils, eosinophils, and monocytes to the site of inflammation. It has been reported that eotaxin 2 induced apoptosis of THP1 cells^43^.

Significantly higher expression of proteins belonging to complement cascade is evident at the proteomic level upon the treatment of cells with TP in comparison with cells treated with NP (Table 1c). The soluble complement proteins detected in plasma are synthesized mainly in the liver, but their local production by circulating immune cells including monocytes is well described^44^.

We documented that the monocytes without contact with physiological cfDNA concentrations in plasma activated emergency mechanisms and began to initiate IIR. The normal functions of these cells were downregulated (notch signaling), potential migration was inhibited, and the genes for attractants of immune cells (*CXCL8* and *CCL24*) were overexpressed (Fig. 2b inset and Fig. 3).

Previously, we found elevated expression of another key member of the cytokine network, namely tumor necrosis factor-alpha (*TNF-α*) in TP treated cells^45,46^. *TNF-α* functions as a master regulator of inflammation and ensures tissue homeostasis^47^. We reported the statistically significant differences validated in qPCR experiments earlier on two different occasions—in our study in which plasma samples of healthy volunteers were used for stimulation of THP1 cells^45^ and in the report studying the stimulatory capacity of plasma samples obtained from patients with celiac disease^46^. Under the stringent conditions set by us for the evaluation of GeneChip experiments, the *TNF-α* was not reported as differentially expressed, but its expression was higher in cells treated with TP per our previous results.

To date, the regulatory mechanisms keeping the cfDNA concentrations in plasma at physiological levels are not well understood. Natural regulatory mechanisms are balancing the NETs production and their clearance. These mechanisms may involve the negative closed feedback loop system, which is widely spread in biology and also used by pharmacologists for drug delivery systems^48^. The failure of the feedback loop mechanism might cause exacerbated DNase I production, resulting in IIR.

Some bacteria are equipped not only with DNases but also with their own 3´-nucleotidases. The activity of these enzymes destructs NETs and converts their degradation products into deoxyadenosine which is toxic namely for macrophages and induces their apoptosis^49^. Different ectonucleotidases are found on the surfaces of different human cells^50,51^, their participation in NETs degradation and toxic product formation cannot be excluded. Our results suggest that the exposition of cells to relatively elevated concentration of cfDNA degradation products can evoke and promote the inflammatory state as the consequence of clearance of high cfDNA concentrations associated with tissue damage^52^ or with the affected clearance of the products of netosis^53^. Elevated deregulated netosis is reported in common comorbidities not only in dialyzed patients^54^ but it is also typical in most comorbidities characterized as risk factors predisposing to serious complications of SARS CoV-2 infection ^16^.

Here we provided *evidence* that the *cfDNA* in human plasma and its clearance represents an *essential* natural *tool for regulation of innate immune response*. We used the THP1 cell line as a model of human monocytes to demonstrate that cfDNA plays an indispensable role in the immune system homeostasis. The cells treated with the native plasma of healthy volunteers expressed genes whose products maintain immune system homeostasis. However, the cells treated with identical plasma samples with degraded cfDNA directly activate IIR with elevated production of mRNA for interleukin 8 at the transcriptomic level. They also upregulated the complement compounds at the proteomic level.

The role of intact and degraded cfDNA and their sensing by cells seems to be one of essential aspects of immune system performance; therefore, further studies focusing on this subject are highly topical. It is of utmost importance to understand the mechanisms of cfDNA release, the clearance and mechanisms of the homeostasis maintenance, as well as the role of different types of cfDNA sequences and their chemical modifications in cfDNA mediated regulatory events, in addition to the recognition of all aspects of cfDNA presence sensed by the cells. The detailed knowledge of mechanisms involved in immune system regulation by the levels of circulating cfDNA may lead to new clinical applications, especially concerning the complete understanding of the pathogenesis of sepsis, COVID-19 and therapeutic DNase treatment. Monitoring the cfDNA level can serve as an actual tool for early well-advanced diagnoses of several diseases such as cancer, sepsis and COVID-19, as well as labor timing.

This study is the first to show the fundamental role of cfDNA and its clearance in plasma of healthy individuals in the regulation of innate immune response, thus warranting further research in this direction.

## Methods

### Subjects

Plasma donors were selected from healthy volunteers who satisfied the following criteria:

1. They were taking no medication
2. They had no chronic illness
3. They had not recovered from an infectious disease in the last two weeks
4. They were in very good physical and psychical status.

The subset of samples described in our previous study^45^ was utilized for expression arrays experiments. Five women and five men aged between 21 and 64 years with age of (35.8 ± 13.8) year (mean ± standard deviation from 10 samples) were selected for validation of quantitative PCR (qPCR) experiments.

The ethical committee of the 1st Faculty of Medicine of Charles University and General Faculty Hospital in Prague, Czech Republic, approved our study. We obtained informed consent from all study participants. All methods were performed per the relevant guidelines and regulations.

### Preparation of plasma samples

Whole blood was collected into Vacuette tubes containing K3 EDTA (Greiner Bio-One). We separated blood plasma using the following steps: centrifugation rate, time and temperature set to 1,800 rpm, 10 min and 4 °C, respectively; then the transfer of plasma into 2 mL low binding collection tubes and centrifugation set to rate of 14,500 rpm for 5 min to remove the residual cells. Finally, we transferred the plasma into clean 2 mL DNA LoBind tubes (Eppendorf). The samples were stored at a temperature of − 80 °C.

Before cell experiments, the plasma sample thawed and split into two identical aliquots. The first one was used at its native state (NP), and the second one was treated with Turbo DNA-free DNase (Thermo Fisher Scientific) according to the manufacturer’s recommendation (TP). Each 100 μl of the plasma sample was treated with 1 μl Turbo DNase, 7 μl water and 12 μl 10 × Turbo DNase buffer. This mix was incubated at 37 °C for 20 min and then Turbo DNase was inactivated by adding 12 μl DNase inactivation reagent, mixed and incubated at room temperature for 5 min. DNase inactivation reagent was removed by centrifugation at an acceleration force of 10,000 g for 1.5 min and the supernatant was transferred into the new tube.

In order to evaluate the influence of individual protocol steps on the alteration of expression profiles, the following experiments were performed: The plasma sample was handled according to the protocol for DNase Turbo treatment but DNAse Turbo was supplemented by water to recognize the effect achieved by this enzyme. Inactivation buffer was added either immediately or after incubation step to recognize the influence of sample incubation at 37 °C with activation buffer. Identical experiments were performed with DNAse Turbo addition. All these plasma samples were used for stimulation experiments with THP1 cells and expressions of all validated genes was determined as described below.

For determination of cfDNA levels in plasma before and after treatment with Turbo DNA-*free* Kit components, the qPCR at QuantStudio 12 K Flex Real-Time PCR System (Applied Biosystems, USA) was performed using the cfDNA samples isolated by QIAamp Circulating Nucleic Acid Kit. PowerUp SYBR Green PCR Master Mix (Life Technologies, USA) constituted one half of 20 μl reaction, the total cfDNA amount was measured using primers for the gene *36B4*^45^ and the standard curve dilution had range from 5 ng to 0.312 ng per reaction.

### Cell cultivation and stimulation

We used the THP1 cell line for stimulation experiments. After the recovery of cells from a deep-frozen state, we used the protocol published earlier ^45,46^. The cells were stimulated for 3 h in the RPMI 1640 medium (Sigma Aldrich) containing 10% of a plasma sample. The cells were collected after 3 h of cultivation; after centrifugation, the supernatant was removed, and the cells were preserved in lysis solution (Sigma-Aldrich) and stored at − 80 °C.

### RNA isolation and reverse transcription

#### RNA isolation and RT for microarray experiments

RNA for qPCR validation was isolated from THP1 cells stored in Lysis Solution after paired stimulation experiments with NP and TP using GenElute Mammalian Total RNA Miniprep Kit (Sigma-Aldrich) according to the protocol provided by the manufacturer and stored at − 20 °C. The reverse transcription was conducted on the thawed sample and performed using the High Capacity cDNA Reverse Transcription Kit (Applied Biosystems) according to the protocol provided by the manufacturer.

### Microarray experiments—transcriptome analysis

The quality and integrity of the total RNA amount were evaluated using the Agilent 2100 Bioanalyzer system (Agilent). Only samples surpassing the minimal quality threshold for the RNA integrity number (also known as RIN) > 7.5 were used in the subsequent transcriptomic assessment. Microarray experiments were performed using the GeneChip Human Gene 2.1 ST Array Strip (Thermo Fisher Scientific) on the GeneAtlas system (Thermo Fisher Scientific) according to the manufacturer’s instructions. The quality control of the chips was performed using the Affymetrix Expression Console (Thermo Fisher Scientific); Transcriptome Analysis Console 4.0 (Life Technologies) was used for subsequent data analysis.

- The current data are available in the ArrayExpress database: no. E-MTAB-8574
- Experiment Title: Exposure of THP1 cells to plasma containing or lacking cfDNA

### qPCR experiments

The set of twelve TaqMan Gene Expression Assays was used to validate the results of microarray experiments: TXNIP (Hs01006900_g1), ARRDC4 (Hs00411771_m1), DDIT3 (Hs00358796_g1), SESN2 (Hs00230241_m1), IRF1 (Hs00971965_m1), HERPUD1 (Hs01124269_m1), HES1 (Hs00172878_m1), MTSS1 (Hs00207341_m1), GPR183 (Hs00270639_s1), CCL24 (Hs00171082_m1), CXCL8 (Hs00174103_m1) and the housekeeping gene PGK1 (Hs99999906_m1) (Applied Biosystems). The reactions were set in 96 well plates (in triplicates) and performed at QuantStudio 12 K Flex Real-Time PCR System (Applied Biosystems); TaqMan Gene expression master mix (Applied Biosystems) was used according to manufacturer’s instructions. The results were multiplied by a factor of 10^3^ to increase resolution and presented in arbitrary units (AU). Statistical analysis was done in GraphPad Prism 5.00.288 software (Graph-Pad Software). First, we performed the D´Agostino-Pearson normality test. In the case of parametric data distribution, the t-test was used; the Wilcoxon matched-pairs signed-rank test was applied for non-parametric data distribution. The statistical significance was set to level ≤ 0.05 for all comparisons.

### Sample preparation for mass spectrometry

Frozen THP-1 cell pellets containing ≈ 500,000 cells were resuspended in 100 μL p phosphate-buffered saline and lysed using buffer composed of 1% sodium dodecyl sulfate (Sigma-Aldrich) in 50 mM 4-(2-hydroxyethyl)-1-piperazineethanesulfonic acid buffer with pH 8.5 (Carl-Roth) supplemented with 1× Complete Ultra Protease Inhibitor Cocktail-ethylenediaminetetraacetic acid (Roche) and shortly sonicated. Mixtures were heated for 5 min at 95 °C and subsequently cooled by placing samples on ice. Twenty-five units of benzonase nuclease (Sigma-Aldrich) was added into each tube, and the tubes were incubated at 37 °C for 30 min to degrade chromatin. The obtained protein mixtures were centrifuged to remove cell debris, and the supernatant was determined by the BCA method using the NanoDrop UV–Vis spectrophotometer (Thermo Fisher Scientific).

Proteins in the lysate (25 μg) were reduced in the Protein LoBind Tubes (Eppendorf) by the addition of dithiothreitol solution (Sigma-Aldrich) to a final concentration of 10 mM and incubated for 30 min at 45 °C. Alkylation was performed by the addition of iodoacetamide to a final concentration of 40 mM and incubation for 30 min at 25 °C in the absence of ambient light. Reactions were quenched by the addition of 1 μL of 1 M dithiothreitol per tube. Then the protein solution was acidified by 1% formic acid to reach pH value between 2 and 3. In the same tube, 50 μg of suspension of paramagnetic carboxylate-modified microparticles Sera-Mag SpeedBeads 1 μm (GE Healthcare) was resuspended in the protein sample solution. Acetonitrile was immediately added to obtain fifty percent solution, and sample suspension was mixed and incubated for 20 min at 25 °C. Next, the tube was placed into a magnetic stand, and paramagnetic microparticles with captured proteins were washed twice using 1 mL of 70% ethanol for 30 s. Finally, microparticles were dried by 200 μL of acetonitrile for 15 s and resuspended in ≈ 50 μL of ≈ 50 mM triethylammonium bicarbonate buffer pH 8.0. Proteins were eluted by on-bead digestion after the addition of trypsin/Lys-C protease mixture (Promega) in enzyme to substrate ratio of 1:35 and overnight incubation at 37 °C^55^. The next day, the obtained peptide eluate was discharged from microparticles, which were again washed twice using 25 μL of 25 mM triethylammonium bicarbonate pH 8.0. The pooled peptide mixture was acidified by 4 μL of 10% trifluoroacetic acid and then desalted using OMIX C18 pipette tips (Agilent) according to the user manual. The clean peptide sample was evaporated on the vacuum concentrator (Eppendorf) and stored at − 80 °C in Protein LoBind tubes.

### nanoLC-MS analysis

Nano reversed-phase columns (EASY-Spray column, 50 cm × 75 µm ID, PepMap C18, 2 µm particles, 10 nm pore size) were used for liquid chromatography/mass spectrometry analysis. Mobile phase buffer A and B was 0.1% formic acid in water and acetonitrile, respectively. Samples were loaded onto the trap column model C18 PepMap100 with 5 μm particle size and dimension of 300 μm × 5 mm (Thermo Scientific) for 4 min at 17.5 μl min^−1^. The loading buffer was composed of water, 2% acetonitrile and 0.1% trifluoroacetic acid. Peptides were eluted with the mobile phase B gradient from 4 to 35% B in 120 min. Eluting peptide cations were converted to gas-phase ions by electrospray ionization and analyzed on a thermo orbitrap fusion modelQ-OT-qIT (Thermo Fisher Scientific). Survey scans of peptide precursors from 350 to 1400 m z^−1^ were performed at 120 K resolution at 200 m/z with a 5 × 10^5^ ion count target. Tandem MS (MS^2^) was performed by isolation at 1.5 Th with the quadrupole, HCD fragmentation with a normalized collision energy of 30, and rapid scan MS^2^ analysis in the ion trap. The MS^2^ ion count target was set to 10^4^, and the maximum injection time was set to 35 ms. Only those precursors with charge states from 2 to 6 were sampled for MS^2^. The dynamic exclusion duration was set to 45 s with a 10 ppm tolerance around the selected precursor and its isotopes. Monoisotopic precursor selection was turned on. The instrument was run in top speed mode with 2 s cycles^56^.

### MS^2^ data analysis

All data were analyzed and quantified with the MaxQuant software (version 1.6.1.0)^57^. The false discovery rate (FDR) was set to 1% for both proteins and peptides, and we specified a minimum peptide length of seven amino acids. The Andromeda search engine was used for the MS^2^ spectra search against the human as downloaded from current Uniprot human database. Enzyme specificity was set as C-terminal to Arg and Lys, also allowing cleavage at proline bonds and a maximum of two missed cleavages. Dithiomethylation of cysteine was selected as fixed modification and N-terminal protein acetylation and methionine oxidation as variable modifications. The *match between runs* feature of MaxQuant was used to transfer identifications to other LC-MS^2^ runs based on their masses and retention time with a maximum deviation of 0.7 min; this was also used in quantification experiments. Quantifications were performed with a label-free algorithm in MaxQuant41^58^. Data analysis was performed using Perseus 1.6.2.2. software^59^.

### Bioinformatic analysis

The sets of differentially transcribed genes and differentially expressed proteins were analyzed using Reactome dababase^60^. The sets were also subjected to Ingenuity Pathway Analysis (Qiagen)^61^.

## Supplementary information

Supplementary Tables.

Supplementary Figure 1.
