# Supplementary figures and images for: Cell-free DNA in plasma as an essential immune system regulator
Source: Sci Rep. 2020 Oct 15;10:17478. doi: 10.1038/s41598-020-74288-2 (PMC7566599; doi:10.1038/s41598-020-74288-2)

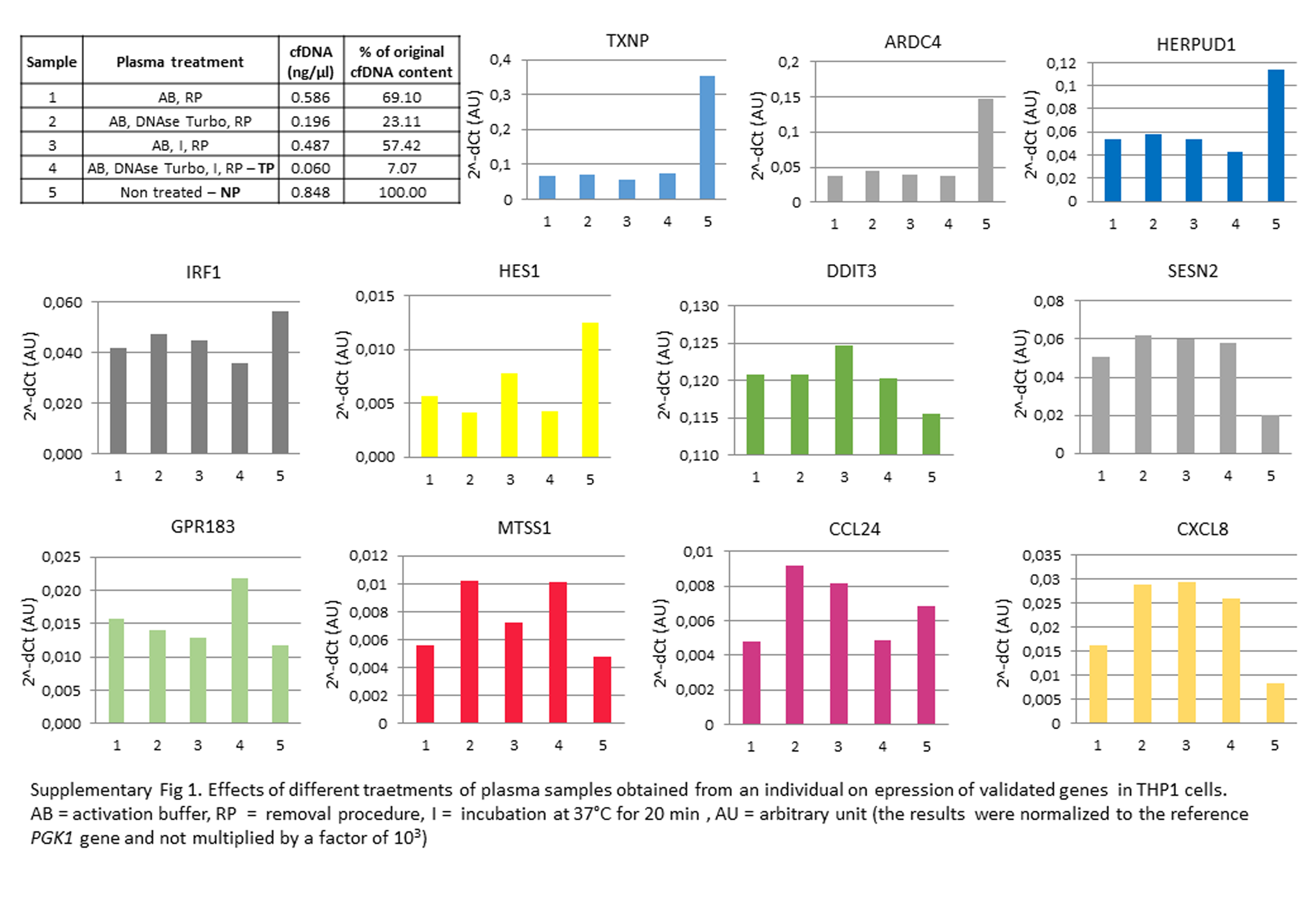

Supplement: Supplementary file 2 — Supplementary Figure 1. [file 41598_2020_74288_MOESM2_ESM.tif]
